# Functionalized Multiwalled CNTs in Classical and Nonclassical CaCO_3_ Crystallization

**DOI:** 10.3390/nano9081169

**Published:** 2019-08-15

**Authors:** Andrónico Neira-Carrillo, Patricio Vásquez-Quitral, Marianela Sánchez, Masoud Farhadi-Khouzani, Héctor Aguilar-Bolados, Mehrdad Yazdani-Pedram, Helmut Cölfen

**Affiliations:** 1 Department of Biological and Animal Sciences, Faculty of Veterinary and Animal Sciences, University of Chile, Santiago P.O. Box 2–15, Chile; 2 Instituto de Ciencias Químicas Aplicadas, Facultad de Ingeniería, Universidad Autónoma de Chile, San Miguel, Santiago 8900000, Chile; 3 Physical Chemistry, Department of Chemistry, University of Konstanz, 78464 Konstanz, Germany; 4 Faculty of Chemical and Pharmaceutical Sciences, University of Chile, Santiago, S. Livingstone 1007, Chile

**Keywords:** multiwalled carbon nanotubes, calcium carbonate, gas diffusion crystallization, amorphous calcium carbonate, calcite, vaterite, prenucleation cluster, mineralization

## Abstract

Multiwalled carbon nanotubes (MWCNTs) are interesting high-tech nanomaterials. MWCNTs oxidized and functionalized with itaconic acid and monomethylitaconate were demonstrated to be efficient additives for controlling nucleation of calcium carbonate (CaCO_3_) via gas diffusion (GD) in classical as well as nonclassical crystallization, yielding aragonite and truncated calcite. For the first time, all amorphous calcium carbonate (ACC) proto-structures, such as proto calcite-ACC, proto vaterite-ACC and proto aragonite-ACC, were synthesized via prenucleation cluster (PNC) intermediates and stabilized at room temperature. The MWCNTs also showed concentration-dependent nucleation promotion and inhibition similar to biomolecules in nature. Incorporation of fluorescein-5-thiosemicarbazide (5-FTSC) dye-labeled MWCNTs into the CaCO_3_ lattice resulted in fluorescent hybrid nanosized CaCO_3_. We demonstrate that functionalized MWCNTs offer a good alternative for controlled selective crystallization and for understanding an inorganic mineralization process.

## 1. Introduction

Multiwalled carbon nanotubes (MWCNTs) are the subject of intense research because of their extraordinary electrical, mechanical, and thermal properties [1]. MWCNTs qualify as suitable nanomaterials for the manufacturing of a variety of composites and organic–inorganic hybrid materials [2]. The use of functionalized MWCNTs for controlling crystal growth and nucleation of inorganic materials, e.g., calcium salts, is a fast-growing research field [3]. Up to now, functionalized MWCNTs have been used as additives for classical crystallization of calcium carbonate (CaCO_3_), showing their ability to control the morphology of and to stabilize different crystalline phases [4,5,6,7]. CaCO_3_ is a natural, biocompatible, and cheap mineral with abundant industrial and biomedical applications, such as drug delivery, scaffolds for regenerative medicine, disease diagnosis, treatment, theranostics, etc. [8]. Additives and templates have been widely used as crystal growth modifiers for controlled classical crystallization [9]. The role of additives in in vitro CaCO_3_ crystallization and nucleation inhibition has been widely reported [10,11,12]. 

On the other hand, proto-structures of additive-free amorphous calcium carbonate (ACC) obtained via the prenucleation cluster (PNC) pathway by controlling temperature and pH have also been described [13]. These proto-structures are interesting because they can potentially predetermine the crystalline polymorph; a strategy also found in biomineralization [14,15]. Despite the interest in nano-bioinorganic materials [16], it had not been possible to generate all ACC proto-structures (proto calcite-ACC (pc-ACC), proto vaterite-ACC (pv-ACC) and proto aragonite-ACC (pa-ACC)) via the PNC pathway at room temperature under the noncrystallization concept [17]. 

Herein, we report for the first time the capability of functionalized MWCNTs to be additives for the simultaneous modification and stabilization of polymorphs and proto-structures of ACC through in vitro crystallization via gas diffusion (GD) and the PNC pathway at room temperature.

## 2. Materials and Methods 

### 2.1. Materials 

MWCNTs (Baytubes^®^ 150 CP) were obtained from Bayer, Leverkusen, Germany. 2-Methylenebutanedioic acid (itaconic acid, IA) was purchased from Sigma-Aldrich (St. Louis, MO, USA). Monomethyl itaconate (MMI) was synthesized by direct esterification of itaconic acid (IA) with methanol or octadecyl alcohol and the purity was checked by Proton nuclear magnetic resonance (^1^H-NMR) [18]. HNO_3_, H_2_SO_4_, and p-toluenesulfonic acid monohydrate were purchased from Sigma-Aldrich. Calcium chloride, ethanol, and hydrochloric acid were obtained from Merck. Ammonium hydrogen carbonate was purchased from J.T. Baker (Phillipsburg, NJ, USA). Ultrapure water (18.2 MΩ) from a LaboStarTM 4-DI/-UV water system was used for all solutions involved in the CaCO_3_ crystallization and zeta potential measurements. Fluorescein-5-thiosemicarbazide (5-FTSC), used as fluorescent dye, was provided by Invitrogen^®^ (Waltham, MA, USA). Glassware cleaning was performed by washing with neutral detergent, rinsing with ultrapure water, sonicating in cold ethanol for 5 min, rinsing with ultrapure water, submerging three times in piranha solution, rinsing again with ultrapure water, washing with acetone, and drying in an oven at 20 °C under vacuum. The piranha solution was prepared by mixing equal parts of H_2_O, HNO_3_, and H_2_O_2_ solutions (1:1:1, *v*/*v*/*v*).

### 2.2. Oxidation and Functionalization of MWCNTs

Oxidized MWCNTs (MWCNT-Ox) were synthesized by using the method reported by Avilés et al. [19]. This method was selected due to the nonaggressive treatment, which results in obtaining MWCNT-Ox with a low degree of structural defects. Briefly, 0.5 g of the pristine MWCNTs were sonicated for 10 min and then mixed with 40 mL of 58% HNO_3_ and 20 mL of 98% H_2_SO_4_ in a 500 mL round-bottomed flask. This mixture was refluxed for 1 h at 140 °C. Then, MWCNT-Ox was washed with 500 mL of H_2_O, and the suspension was filtered, washed with distilled water, and dried under vacuum at 60 °C for 24 h. For functionalization reactions, 0.2 g of MWCNT-Ox was dispersed in 20 mL of acetone in an ultrasonic cleaning bath for 5 min. Then, the MWCNT-Ox suspension was mixed with 2 g of with IA or MMI together with 80 mL of acetone in a round-bottomed flask. Subsequently, 0.3 g of p-toluenesulfonic acid monohydrate was added and refluxed for 3 h. Both functionalized MWCNTs were separated by filtration under vacuum, washed with acetone to remove the excess of unreacted MMI or IA, and dried under reduced pressure at 70 °C for 24 h. Finally, MWCNT-IA and MWCNT-MMI were used as templates for CaCO_3_ crystallization.

### 2.3. Classical Crystallization of CaCO_3_

Classical crystallization of CaCO_3_ in the presence of oxidized or functionalized MWCNTs was carried out using the GD method. Pristine MWCNTs were used as control. GD crystallization was performed as described previously [20,21,22]. All experiments were carried out inside Petri dishes, by using 1.0 mg/mL of MWCNTs at 20 °C for 24 h. Moreover, a negative-control crystallization without additive was also performed. For these experiments, 1.6 mg of each type of MWCNTs dispersed in deionized water was prepared and used as stock. Moreover, CaCO_3_ crystallization was carried out with MWCNT samples at a concentration of 1.0 mg/mL at pH 9.0 and 20 °C for 24 h using homemade glass macro-bridges, fabricated by a glass craftsman by cutting a 1.6 diameter assay tube. These recipients have a total volume of 1.5 mL including 5 μL of buffered 200 mM CaCl_2_ solution according to the GD method. 5-FTSC, which can bind to carboxylate, aldehyde, and ketone groups, was also utilized as a novel strategy to visualize the role of oxidized and functionalized MWCNTs as modulating templates for CaCO_3_ crystallization. 

### 2.4. Nonclassical Crystallization of CaCO_3_

Prenucleation cluster (PNC) experiments for the nonclassical crystallization of CaCO_3_ were carried out in the presence of oxidized and both types of functionalized MWCNTs as additives as follows. All experiments were carried out by using pristine MWCNT and the functionalized MWCNTs at 1–20 mg/mL at constant pH 9.0 and 20 °C. The control PNC experiment was performed in the absence of MWCNTs. For all PNC assays, a stock suspension of 1.6 mg of each MWCNT template in deionized water was prepared. For this, 10 mM CaCl_2_ solution was dosed at a constant rate of 0.01 mL/min to MWCNTs dispersed in 20 mL of 10 mM carbonate buffer under constant stirring at 800 rpm. A constant pH was maintained by automatic counter-titration using 10 mM NaOH solution. Calibration and reference experiments were performed by dosing 10 mM CaCl_2_ in water and carbonate buffer (10 mM, 20 mL) at different pH values. The CaCO_3_ nucleation onset was identified by a sudden increase in the NaOH consumption. The crystallization reactions were quenched in an excess of absolute ethanol before the nucleation. Then, ACC particles were isolated by decantation and centrifugation at 9000 rpm for 10 min, followed by washing cycles with ethanol and acetone in order to remove remaining water. ACC entities were identified by Fourier transform infrared spectroscopy (FTIR) spectroscopy. A similar experimental protocol has been reported and utilized [23].

### 2.5. Spectroscopic, SEM, and TEM Analyses of MWCNTs and CaCO_3_ Crystals

**Figure 1:**
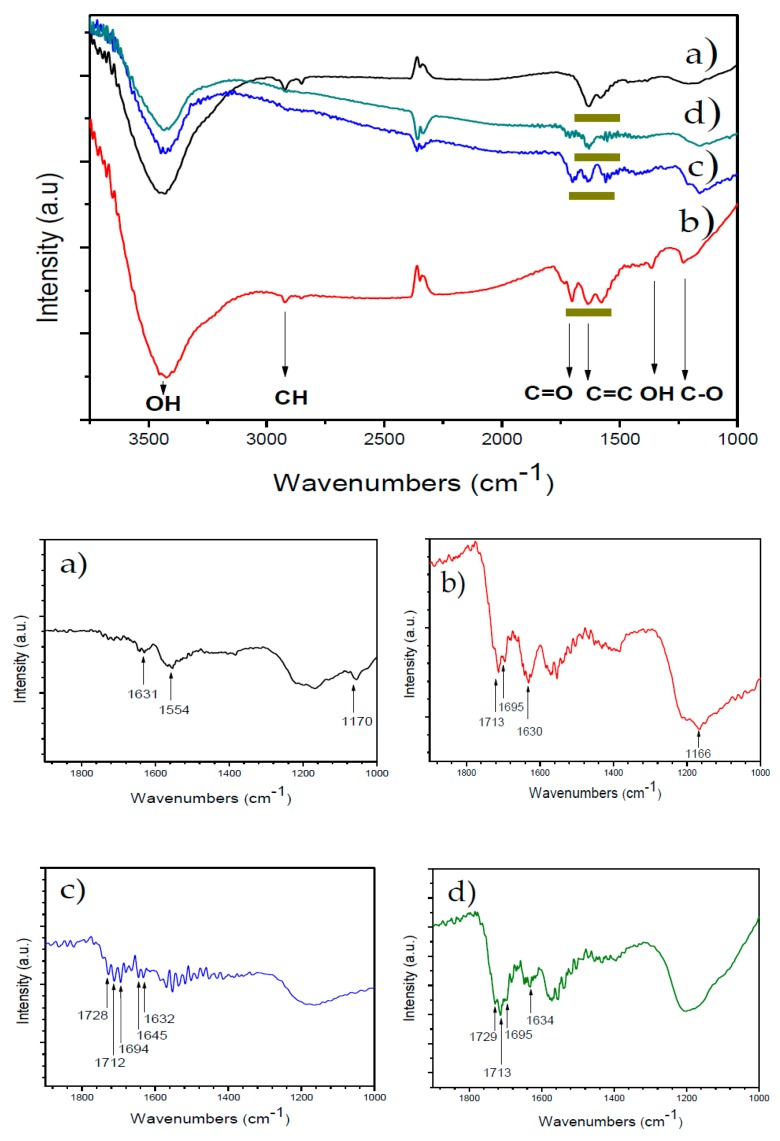
FTIR of oxidized and functionalized multiwalled carbon nanotubes (MWCNTs): (**a**) MWCNT, (**b**) MWCNT-Ox, (**c**) MWCNT-IA, and (**d**) MWCNT-MMI. The zones marked in dark yellow represent each MWCNT additive.

The presence of hydroxyl and carboxyl functional groups in the oxidized MWCNT (MWCNT-Ox) and both types of functionalized MWCNTs—with itaconic acid (MWCNT-IA) and monomethylitaconate (MWCNT-MMI)—was confirmed by FTIR (Figure 1a–d). The TEM images of the CaCO_3_ crystals were obtained with a Philips Tecnai 12 Bio Twin 120 K transmission electron microscope (TEM) (Philips Tecnai 12 Bio Twin, Eindhoven, the Netherlands) (Figure S1). FTIR spectra of MWCNTs and sediment of ACC particles were taken by using an Interspec p/n 200-X and a Perkin Elmer spectrum 100 FT-IR spectrometers (Interspectrum OU, Toravere, Estonia), respectively. The FTIR of ACC sediment was taken after PNC experiments were stopped, 500 s before the nucleation onset. PNC assays were performed by using the highest concentration of MWCNT templates. 

The surface morphology and microanalysis of the resultant CaCO_3_ crystals were observed by scanning electron microscopy (SEM). The SEM analysis of CaCO_3_ crystals was carried out by using a SEM TESLA BS 343 A microscope (TESCAN, Brno, Czech Republic). Once the microbridges were dried, crystal particles were covered with gold using a sputter coater instrument (EMS-550). The MWCNTs labeled with 5-FTSC were observed using an optical Nikon eclipse E400 microscope coupled to a computer with morphometric software (Image Pro-Plus, Media Cybernetics, Melville, NY, USA). Optical images were obtained in a digital camera at ×40 magnification and an exposure time of 20 ms to 2 s with a resolution of 2560 × 1920 pixels. TEM and SEM of CaCO_3_ before and post-nucleation point during PNC assays in the presence of MWCNT and MWCNT-Ox were performed by using JEOL/JEM 1200 EX II and FEI Inspect F50 instruments, respectively. 

### 2.6. Zeta Potential of Oxidized and Functionalized MWCNTs

Oxidized zeta potential measurements were performed in a Brookhaven potential analyzer (Brookhaven Instruments Corp., Holtsville, NY, USA). Ultrasonication of suspensions of pristine MWCNT, MWCNT-Ox, MWCNT-IA, and MWCNT-MMI was performed before both determinations. MWCNTs suspensions were prepared in 1 mM KCl solution at different pH (4.0, 7.0, and 9.0) and then sonicated. Sonication and ultrasonication of MWCNT samples were performed with an ultrasonic cleaning bath Power Sonic 410 and Branson S-450 digital sonifier equipped with a microtip, respectively. Different sonication cycles (up to three) were performed for all MWCNTs. Cycles 1 and 2 consisted in 2 min of sonication, then 1 min of ultrasonication was applied to MWCNT through a tapered microtip. In the third cycle, 10 min of ultrasonication was applied. For all ultrasonication, the applied amplitude was 40%. For zeta potential and particle size determinations of MWCNTs, 10 μL of MWCNT stock suspensions was taken with a Gilson Pipetman Classic 10 μL (P10) and mixed with 10 mL of bifiltered 1 mM KCl at pH 4.0, 7.0, and 9.0. For this, 50 μL of each MWCNT suspension was mixed with 5 mL of 1 mM KCl solution, also prepared at these pH values, to obtain a final concentration of 1.6 × 10^−5^ mg/mL of all MWCNT samples. 

### 2.7. Back Titration of COOH Groups of MWCNTs

The concentration of –COOH groups of oxidized and functionalized MWCNTs were determined by automatic titration. Briefly, MWCNTs were added to 50 mL of 0.01N NaOH solution and left to stir overnight at room temperature. The resultant mixture was back-titrated with a 0.01 N HCl solution.

## 3. Results and Discussion

### 3.1. Characterization of MWCNTs 

Characterization of MWCNT additives was performed by using FTIR and TEM techniques; these results confirmed the surface chemical modification of MWCNTs and that the functionalized MWCNTs are less aggregated than the pristine MWCNT, respectively (Figure 1 and Figure S1). The FTIR spectrum of the pristine MWCNT (Figure 1a) shows an absorption band centered at 3425 cm^−1^ corresponding to O–H groups of adsorbed water. The absorption bands due to deformation vibrations of the hydroxyl groups of MWCNT-Ox and those at 1635 cm^−1^ and 2920 cm^−1^ are assigned to the stretching vibrations of C=C and C–H bonds, respectively (Figure 1b). Figure S1b shows new absorption bands in comparison to the FTIR of the pristine MWCNTs. The absorption bands at 3430 cm^−1^ and 1365 cm^−1^ are assigned to stretching and bending vibrations of the O–H bond, and the bands at 1702 cm^−1^ and 1230 cm^−1^ are assigned to the C=O and C–O stretching of carboxyl groups, confirming the presence of hydroxyl and carboxyl groups. The FTIR spectrum of MWCNT-IA (Figure 1c) shows additional absorption bands in the region of 1700 cm^−1^ due to the stretching vibrations of carboxyl groups. Moreover, new absorption bands in the spectrum of Figure S1c are observed at 1630 cm^−1^ due to stretching vibrations of vinyl C=C bonds of IA as well as at 1400 cm^−1^ due to bending vibrations of O–H and at 1162 cm^−1^ to stretching vibrations of C–O bonds. The FTIR spectrum of MWCNT-MMI (Figure 1d) shows absorption bands at 1735 cm^−1^ and 1711 cm^−1^, indicating the presence of C=O groups corresponding to the ester and carboxylic acid, respectively. Moreover, absorption bands at 1400 cm^−1^ and 1162 cm^−1^ corresponding to bending vibrations of O–H and stretching vibrations of C–O bonds are also observed. Zeta potentials of the pristine, oxidized, and functionalized MWCNTs at room temperature and pH 7.4 before the sonication are in the range of −1.62 and −6.20 mV (Figure S2 and Table S1). The polar surface of MWCNTs may attract Ca^2+^ ions before and during the nucleation phase, indicating a preferred interaction site during nucleation, and crystal growth of CaCO_3_ with a similar manner to biomolecules in biogenic minerals. The hydrodynamic diameters of oxidized and functionalized MWCNT dispersed in aqueous 1 mM KCl solution were decreased after three sonication cycles (Figure S3 and Table S2). Here, smaller particle sizes were obtained at higher pH values. The average particle sizes of all functionalized MWCNTs were smaller than those of pristine MWCNT, with that of MWCNT-IA being the smallest at pH 9.0. The sonication process affected the particle size distribution of all MWCNTs dispersed in water at different pH values (Figure S4). Independent of the pH of the suspension, the polydispersity of all MWCNTs decreased after the first sonication cycle, while no significant differences were detected after the second and third sonication cycles. 

Table 1 shows the zeta potential values of all MWCNTs after the third sonication cycle in 1 mM KCl solution at pH 4.0, 7.0, and 9.0. It is seen that the zeta potential value at pH 4.0 is negative for oxidized and functionalized MWCNTs and positive for pristine MWCNTs. Moreover, at pH 7.0 and pH 9.0, all MWCNTs show negative zeta potential values, promoting interaction with Ca^2+^ (Table 1). These results show that the increase of negative charge of the MWCNTs will have a strong influence on nucleation inhibition and stabilization in both classical and nonclassical crystallization, as further found by FTIR analysis. For example, the pristine MWCNTs did not influence the crystallization of CaCO_3_ using the GD method. However, they were able to stabilize pa-ACC through the PNC assays.

An automated acid–base titration was performed to determine the concentration of –COOH groups present in all MWCNTs (Figure S5) [24]. The highest concentration of –COOH groups is in the range of 12–15 mmol/g for MWCNT-IA and MWCNT-MMI and the lowest is 1.8 mmol/g for the pristine MWCNTs (Table S3).

### 3.2. Gas Diffusion (GD) as Classical Crystallization

**Figure 2:**
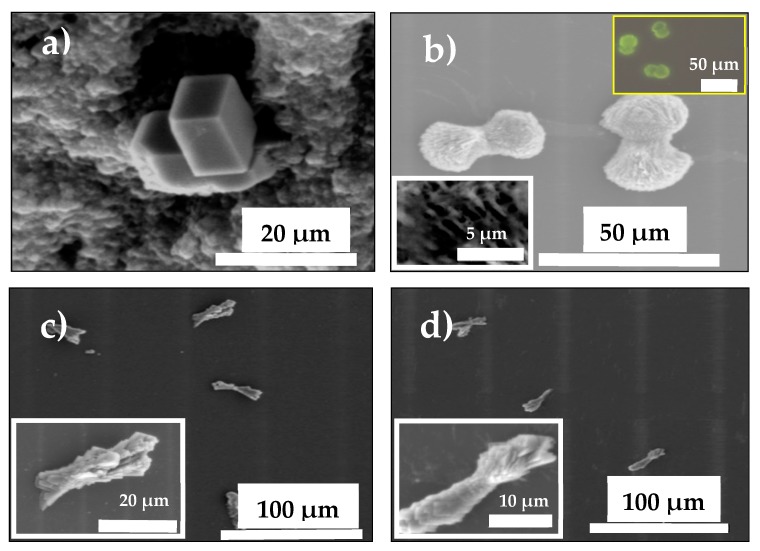
SEM images of CaCO_3_ crystals grown in the presence of MWCNTs: (**a**) MWCNT, (**b**) MWCNT-Ox, (**c**) MWCNT-IA, and (**d**) MWCNT-MMI. Inset in Figure 2b shows an optical image of fluorescent aragonite labeled with 5-FTSC.

A set of GD crystallizations of CaCO_3_ was performed in triplicate at 24 °C over 24 h to evaluate the effect of all MWCNT additives on the nucleation and crystal growth of CaCO_3_ [25,26,27]. As shown in Figure 2a–d, different CaCO_3_ morphologies were obtained in the presence of pristine, oxidized, and functionalized MWCNTs during the crystallization. Figure 2a shows the morphology of typical rhombohedral calcite obtained with pristine MWCNTs (negative control). We observed that MWCNTs did not affect the CaCO_3_ morphology in all GD crystallization assays. However, dumbbell-shaped aragonite crystals with 30 µm diameter were obtained when MWCNT-Ox was used as additive (Figure 2b); similarly dumbbell-shaped calcite was obtained by using poly(ethylene glycol)-block-poly(methacrylic acid) [28]. High-magnification images of these crystals show a triangular microstructure on the surface. Fluorescent aragonite with a similar shape, obtained with a 5-FTSC probe, is seen in the inset in Figure 2b, demonstrating the incorporation of MWCNT-Ox into CaCO_3_ crystals. The same results were observed for all fluorescent hybrid MWCNT–CaCO_3_ particles (Figure S6). Similar labeling behavior was observed for all MWCNTs with slightly varying intensity in the resultant calcite, truncated calcite, and aragonite crystals due to the incorporation of 5-FTSC and functional groups of MWCNT. Figure 2c,d show characteristic and differently oriented CaCO_3_ crystal morphologies resulting from using MWCNT-IA or MWCNT-MMI. The presence of anionic groups in both types of functionalized MWCNTs induces morphological changes of the CaCO_3_ crystals. Our findings also show that these anionic functionalized MWCNTs stabilized the formation of spherical CaCO_3_ nanoparticles (Figure S7). Hybrid MWCNT–CaCO_3_ particles showed a rough surface, suggesting an effective modification capacity of both types of functionalized MWCNTs. A similar morphological effect on the MWCNT–CaCO_3_ particles was also obtained when GD crystallization was performed using glass macro-bridges (Figure S8).

### 3.3. Prenucleation (PNC) as Nonclassical Crystallization

**Figure 3:**
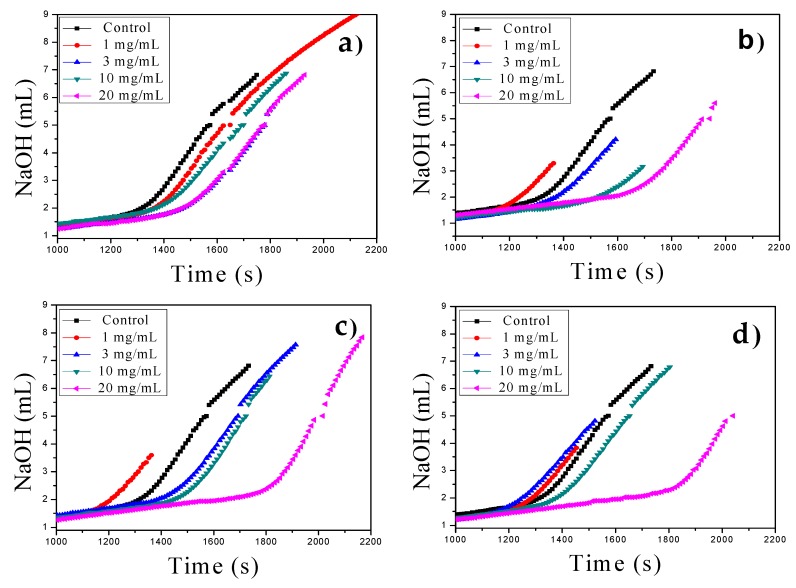
Prenucleation cluster (PNC) assays of CaCO_3_ using MWCNTs in the range of 1–20 mg/mL at constant pH of 9.0 at 20 °C: (**a**) MWCNT, (**b**) MWCNT-Ox, (**c**) MWCNT-IA, and (**d**) MWCNT-MMI.

PNC assays of CaCO_3_ via titration experiments for nonclassical crystallization of CaCO_3_ were also carried out with all functionalized MWCNTs as additives in a similar manner as previously reported [29,30]. The MWCNTs were tested at different concentrations (1–20 mg/mL) at constant pH of 9.0 at 20 °C in the titration assays showing concentration-dependent nucleation behavior, evident from a slope change in all titration curves (Figure 3). The control PNC experiment was performed in the absence of MWCNTs. We found that at higher concentrations (10–20 mg/mL), all MWCNTs act as nucleation inhibitors. However, at low concentrations, in the range of 1–3 mg/mL, crystal nucleation occurs earlier for all oxidized and functionalized MWCNTs with respect to the reference without additive, and only pristine MWCNT act as a nucleation inhibitor at all concentrations. These findings suggest that the charge density on the surface of oxidized and functionalized MWCNTs, at different concentrations, modifies their additive behavior during the in vitro crystallization as a promoter or inhibitor, in a similar manner to that which occurs in nature, where anionic macromolecules control biological and pathological crystallization.

### 3.4. Stabilization of ACC Proto-Structures

To evaluate the stabilization effect of all MWCNTs on ACC proto-structures, FTIR analysis was performed (Figure S9a,b). Here, MWCNTs showed no crystalline absorption bands of CaCO_3_ as compared with the three polymorphs of CaCO_3_, namely calcite, aragonite, and vaterite, in the region of 680–760 cm^−1^ (Figure S9a). Nevertheless, absorption bands in the amorphous region of 820–910 cm^−1^ ascribed to ACC proto-structures was also observed, which indicate that there is no crystalline material that could have been occluded in the ACC proto-structures or partially crystallized into one of the CaCO_3_ polymorphs (Figure S9b). 

**Figure 4:**
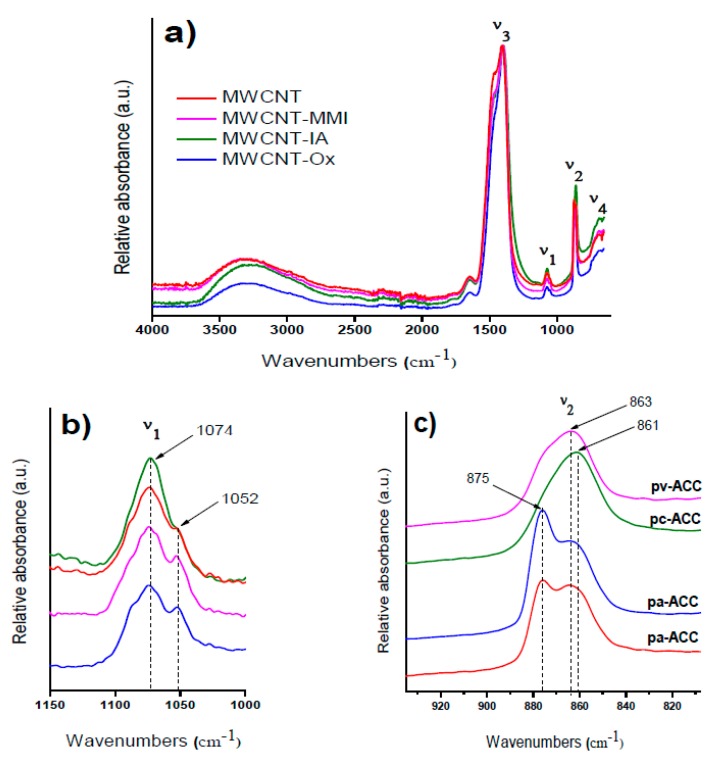
FTIR spectra of amorphous calcium carbonate (ACC) proto-structures obtained in the presence of MWCNT additives: (**a**) full spectra with ν1, ν2, ν3, and ν4 bands; (**b**) zoomed-in view for the ν1 band and (**c**) zoomed-in view for the ν2 band.

In particular, the effect of different MWCNTs on the all amorphous spectra from ν1 to ν4 bands of ACCs formed via quenching the solution before nucleation as is shown in Figure 4a–c. The broad band of water located at 3300 cm^−1^ as well as the absence of a sharp band in the ν4 region indicates that the samples are indeed amorphous (Figure 4a,b). As it was shown before, the best band for differentiating between different ACCs is the ν1 band, which in this case partially overlaps with the C–H vibrations from the MWCNTs at 1052 cm^−1^. Therefore, the more straightforward way to compare the spectra is to refer to the ν2 band. Our results show two stretching absorption bands at 875 cm^−1^ and 863 cm^−1^, corresponding to pa-ACC ascribed to C=O and C–O groups in the presence of MWCNT-Ox (Figure 4c). Surprisingly, pa-ACC was also obtained with pristine MWCNTs containing low concentrations of –COOH at room temperature. This coincides very well with the results found by Gebauer et al. at elevated temperatures [13,31]. The stabilization of pc-ACC and pv-ACC is in accordance with a pronounced carbonate ν1 absorption region and with two stretching absorption bands at 861 cm^−1^ (pc-ACC) and 863 cm^−1^ (pv-ACC) stabilized with MWCNT-IA and MWCNT-MMI, respectively (Figure 4c). Also, the shoulder for pv-ACC at around 875 cm^−1^ can be observed. To the best of our knowledge, this is the first report of all ACC proto-structures being formed at room temperature and ambient pressure. The ACC proto-structures correspond to highly dynamic chemical entities that transform rapidly into crystalline structures, and due to this in the current study, the quantification of the amount of each stabilized ACC proto-structure was not performed.

**Figure 5:**
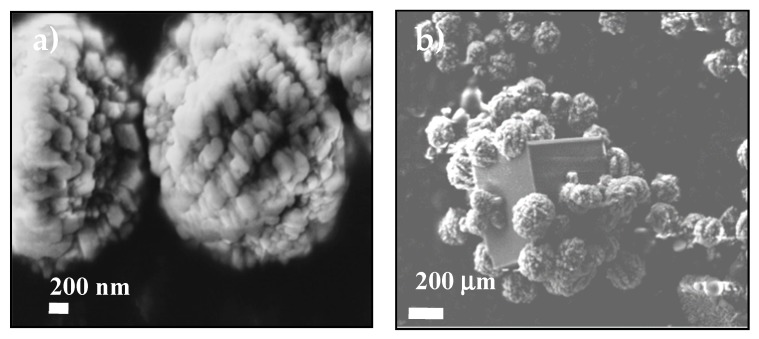
SEM images showing the ACC proto-structures using (**a**) MWCNT-MMI as additive and (**b**) the first calcite crystal surrounded by ACC proto-structures.

**Figure 6:**
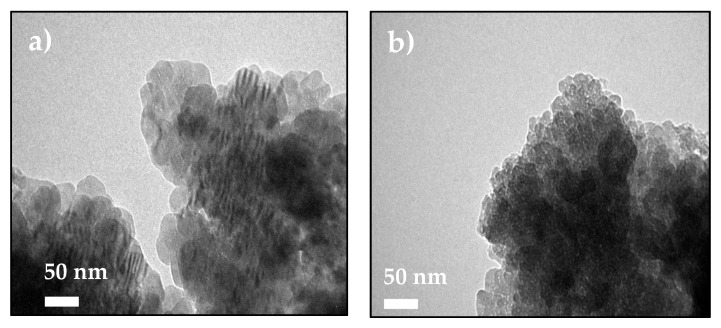
TEM micrographs of ACC proto-structures using (**a**) MWCNT and (**b**) MWCNT-Ox as additive for PNC assays at pH 9.0.

The stabilization of ACC proto-structures and the surrounding crystalline calcite particles were observed by SEM (Figure 5a,b). Figure 5a shows CaCO_3_ beads with 1–3 µm diameter composed of hundreds of nanometric proto-ACC particles obtained with MWCNT-MMI. Figure 5b shows how the first crystalline calcite particles appear after the nucleation stage. In addition, TEM, electron diffraction patterns, and SEM before and after nucleation in PNC assays of CaCO_3_ at pH 9.0 with MWCNT and MWCNT-Ox were also performed (Figure S10). TEM micrographs shows proto-ACC particles obtained with MWCNT and MWCNT-Ox (Figure 6). Vertical bundles of pristine MWCNTs in the ACC proto-structures are clearly seen in Figure 6a, whereas this was not observed for MWCT-Ox (Figure 6b). This is probably associated with a disentanglement of the functionalized MWCNTs.

**Figure 7:**
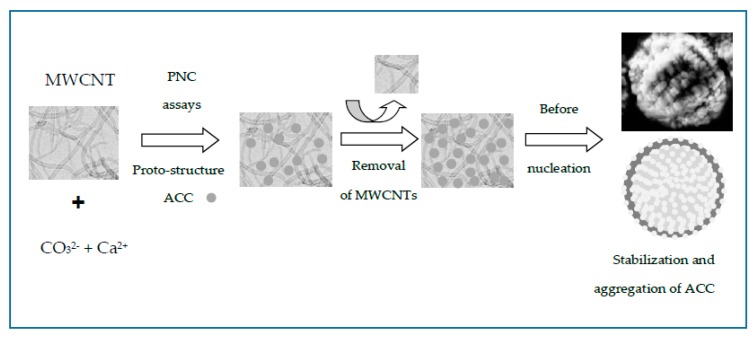
Cartoon of the possible mechanism of the entrapment of MWCNTs into CaCO_3_ beads producing stabilized ACC microparticles.

According to our experimental results, Figure 7 illustrates the cartoon of the possible mechanism of the entrapment of MWCNTs into CaCO_3_ beads during the PNC assays. The nanosized matter was efficiently entrapped by CaCO_3_ during the assembly of hybrid MWCNT–CaCO_3_ microbeads. In addition, TEM images and electron diffraction (ED) patterns of ACC and CaCO_3_ particles obtained in the presence of MWCNTs were obtained (Figure S11a–c). The CaCO_3_ particles showed reduced MWCNT content and crystalline pattern compared to MWCNT–CaCO_3_ microbeads after in situ burning. Our findings show that functionalized MWCNTs can modulate the crystal morphology by face-selective adsorption in GD crystallization and also aggregate nanoparticles in PNC assays, leading to crystals with complex shapes.

## 4. Conclusions

In summary, we firstly demonstrated that the use of functionalized MWCNTs as additives is a good alternative for controlled CaCO_3_ precipitation, by both classical (GD) and nonclassical (PNC) crystallizations. Aragonite and truncated calcite were obtained when GD was performed. This research demonstrated, for the first time, that all ACC proto-structures can be stabilized at 20 °C and ambient pressure in PNC titration assays. The use of functionalized MWCNTs represents an interesting approach to investigate various aspects of controlled CaCO_3_ crystallization, such as polymorphism and stabilization of proto-ACC entities. We believe that oxidized MWCNTs and MWCNTs functionalized with itaconic acid (IA) and IA ester groups, used as additives, on the nucleation, crystal growth, and polymorphs of CaCO_3_ or other calcium salts can be used as morphological modifiers and promoters or inhibitors of biological crystallization. This approach could enable the preparation of bioinspired nanomaterials with advanced and remarkable properties.

## Figures and Tables

**Table 1:** Zeta potential values of all MWCNTs after the third ultrasonication cycle (Ox = oxidized, IA = itaconic acid, MMI = monomethylitaconate).

pH	MWCNT	MWCNT-Ox	MWCNT-IA	MWCNT-MMI
4.0	+9.2 ± 3.5	−24.4 ± 10.5	−22.9 ± 4.1	−22.3 ± 3.0
7.0	−51.4 ± 0.9	−54.7 ± 1.2	−51.0 ± 3.1	−59.5 ± 1.0
9.0	−46.3 ± 0.7	−64.6 ± 1.5	−50.1 ± 4.1	−53.6 ± 3.8

## Supplementary Materials

The following are available online at https://www.mdpi.com/2079-4991/9/8/1169/s1, Figure S1: TEM images of MWCNTs additives such as pristine MWCNTs, MWCNT-Ox, MWCNT-IA, and MWCNT-MMI. Figure S2: Average zeta potential measurements of oxidized and functionalized MWCNTs obtained after 10 runs and sampling time of 128 µs without sonication. Figure S3: Lognormal distribution and effective diameter (nm) of oxidized and functionalized MWCNT obtained after 10 runs at elapsed time of 30 min after the third ultrasonication cycle in aqueous solution at pH 9.0. Figure S4: Particle size distribution (polydispersity) of oxidized and functionalized MWCNT dispersed in nanopure water at pH 4.0, pH 7.0, and pH 9.0. Figure S5: Automatic titration of MWCNT-IA and MWCNT-MMI. Figure S6: Optical microscopy images of fluorescent hybrid MWCNT–CaCO_3_ particles grown in the presence of MWCNTs and 5-FTSC. Figure S7: SEM images of spherical nanoparticles on the CaCO_3_ crystal surface obtained with MWCNT-IA and MWCNT-MMI. Figure S8: SEM images of CaCO_3_ crystals grown in presence of non-sonicated MWCNTs using macro-bridges. Figure S9: FTIR of ACC using MWCNT additives. Figure S10: TEM images and electron diffraction (ED) patterns of ACC proto-structures and SEM images of CaCO_3_ crystals grown in the presence of MWCNT and MWCNT-Ox before and post-nucleation points performed via PNC essays at pH 9.0. Figure S11: TEM images and electron diffraction (ED) patterns of ACC and CaCO_3_ particles in the presence of MWCNT. Table S1: Zeta potential and half-width measurements of oxidized and functionalized MWCNTs obtained after 10 runs and sampling time of 128 µs without sonication. Table S2: Effective diameter, half-width, and polydispersity of oxidized and functionalized MWCNTs obtained after 10 runs at elapsed time of 30 min after the third ultrasonication cycle in aqueous solution at pH 9.0. Table S3: Concentration of carboxylic acid groups of all MWCNTs determined by using acid–base titration.
