# Supplementary material for: Functionalized Multiwalled CNTs in Classical and Nonclassical CaCO3 Crystallization
Source: Nanomaterials (Basel). 2019 Aug 15;9(8):1169. doi: 10.3390/nano9081169 (PMC6723705; doi:10.3390/nano9081169)
Supplement: Supplementary file 1 [file nanomaterials-09-01169-s001.pdf]

SUPPLEMENTARY MATERIAL

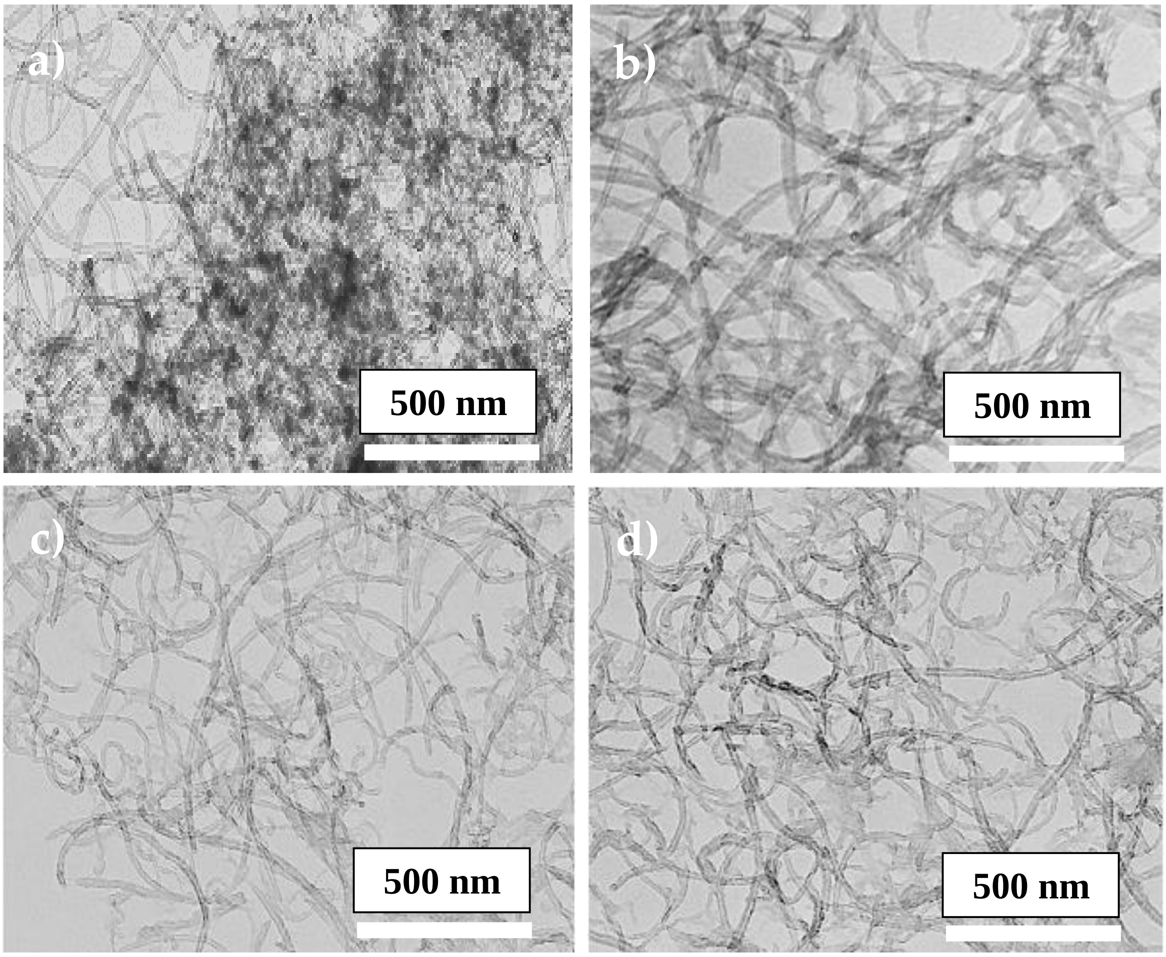

Figure S1. TEM images of MWCNTs additives. a) pristine MWCNT, b) MWCNT-Ox, c) MWCNT-IA and d) MWCNT-MMI.

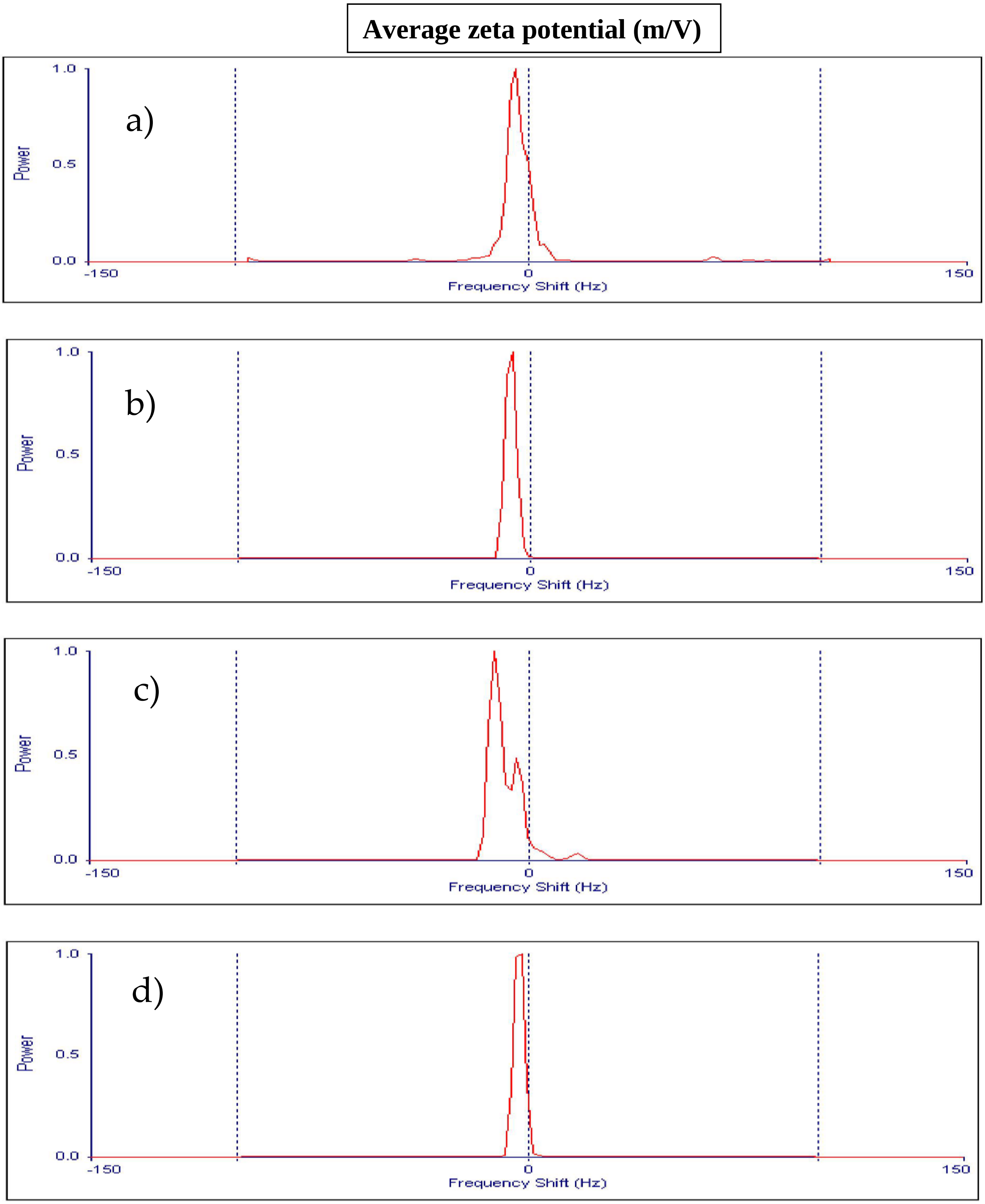

Figure S2. Average Zeta potential measurements of oxidized and functionalized MWCNTs obtained after 10 runs and sampling time of 128 µs without sonication a) pristine MWCNT, b) MWCNT-Ox, c) MWCNT-IA and d) MWCNT-MMI. The dashed line in the graph indicates the zero-frequency shift at 0 mV.

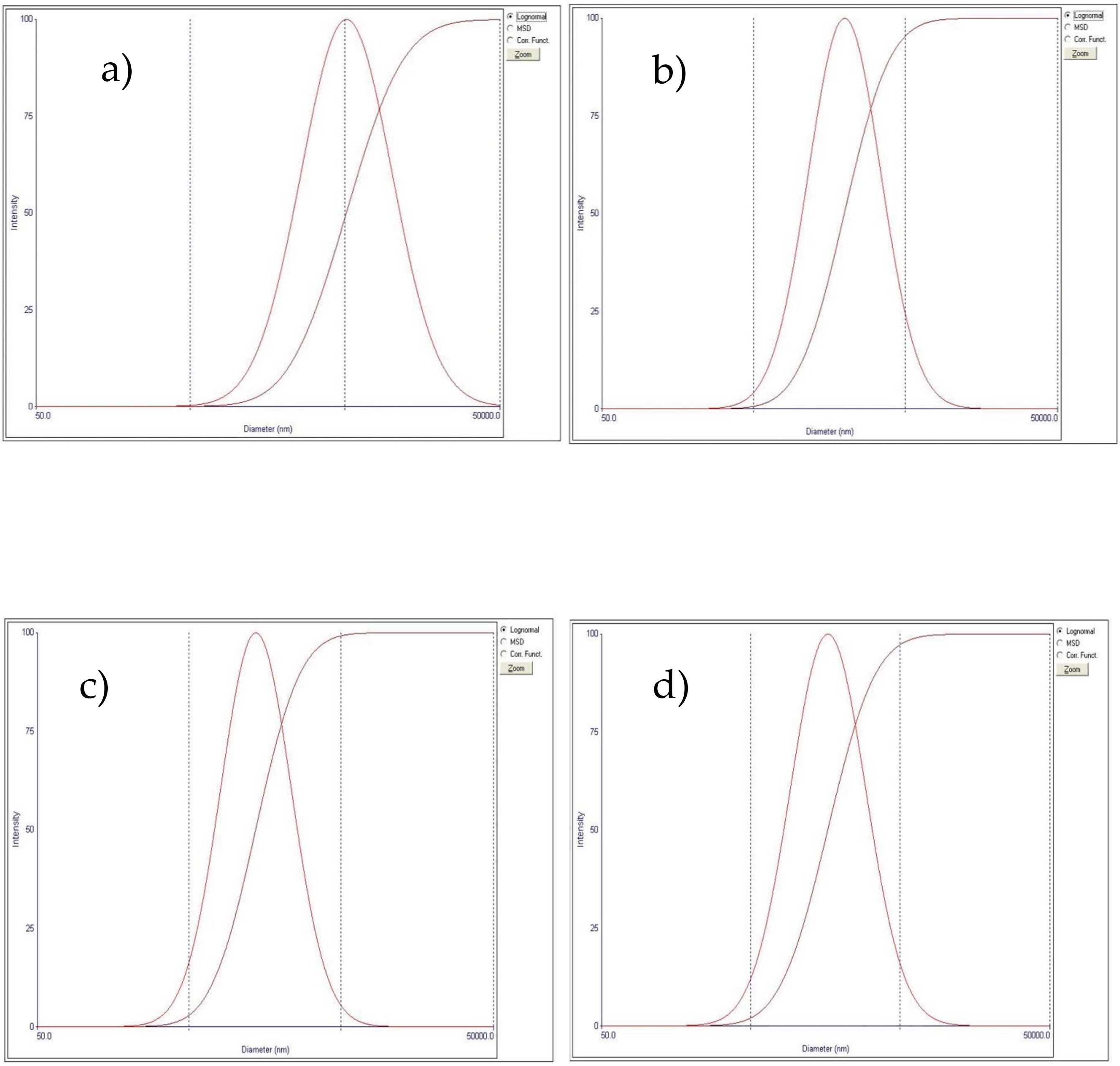

Figure S3. Lognormal distribution and effective diameter (nm) of oxidized and functionalized MWCNT obtained after 10 runs at elapsed time of 30 min after the third ultrasonication cycle in aqueous solution at pH 9.0: a) pristine MWCNT, b) MWCNT-Ox, c) MWCNT-IA and d) MWCNT-MMI.

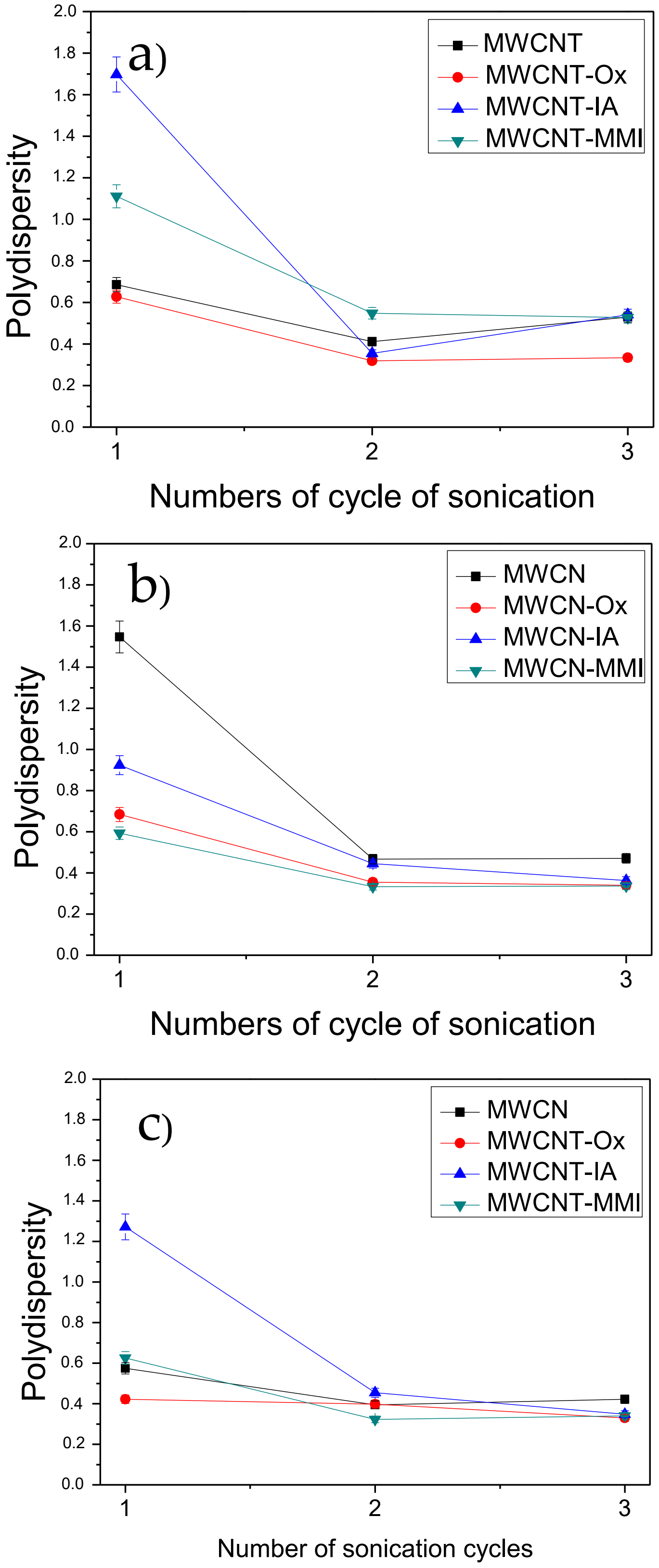

Figure S4. Particle size distribution (Polydispersity) of oxidized and functionalized MWCNT dispersed in nanopure water at pH a) 4.0, b) 7.0 and c) 9.0.

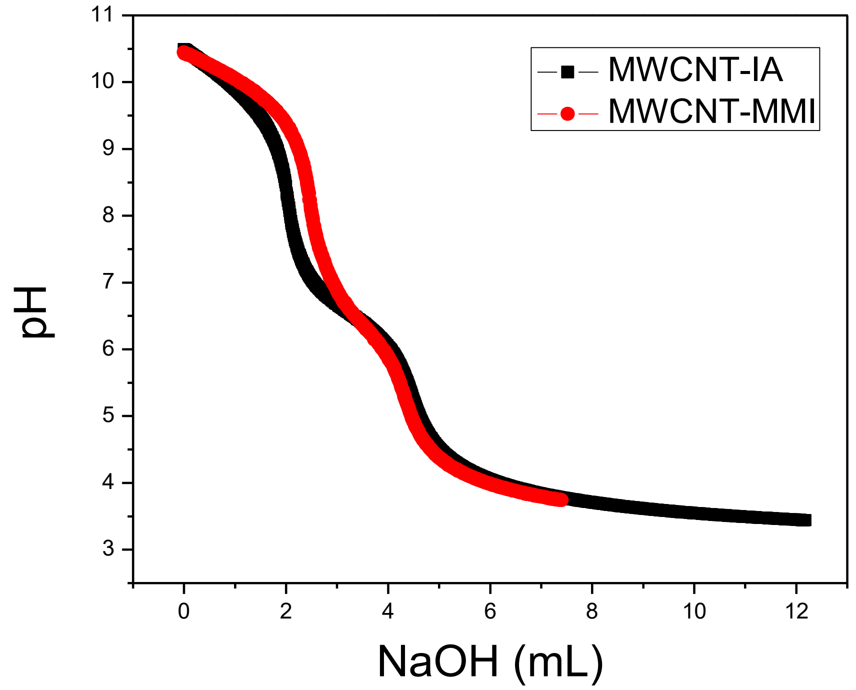

Figure S5. Automatic titration of MWCNT-IA and MWCNT-MMI.

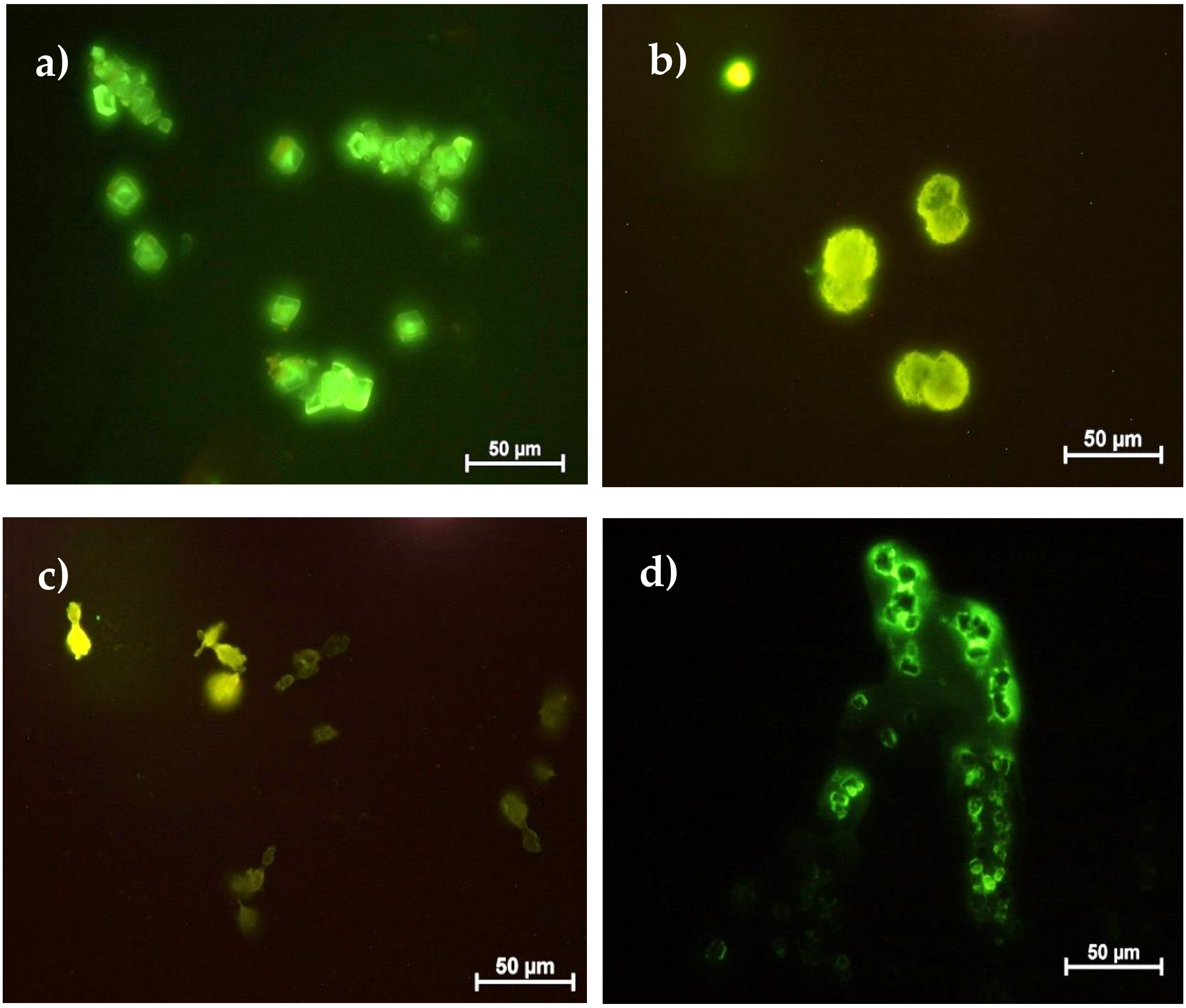

Figure S6. Optical microscopy images of fluorescent hybrid MWCNT-$CaCO_3$ particles grown in the presence of MWCNTs and 5-FTSC. a) pristine MWCNT, b) MWCNT-Ox, c) MWCNT-IA and d) MWCNT-MMI. Pictures were obtained with objective 40X magnification.

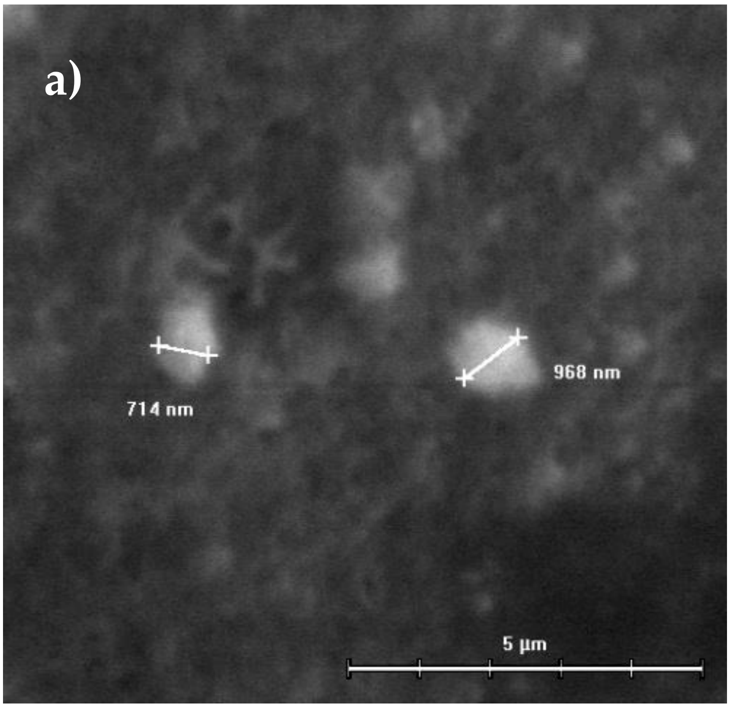

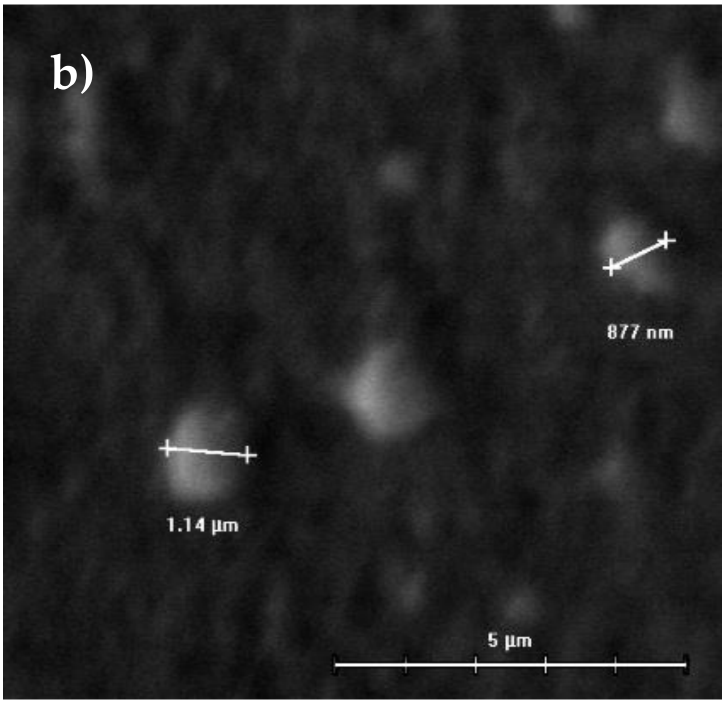

Figure S7. SEM images of spherical nanoparticles on the $CaCO_3$ crystal surface obtained with a) MWCNT-IA and b) MWCNT-MMI.

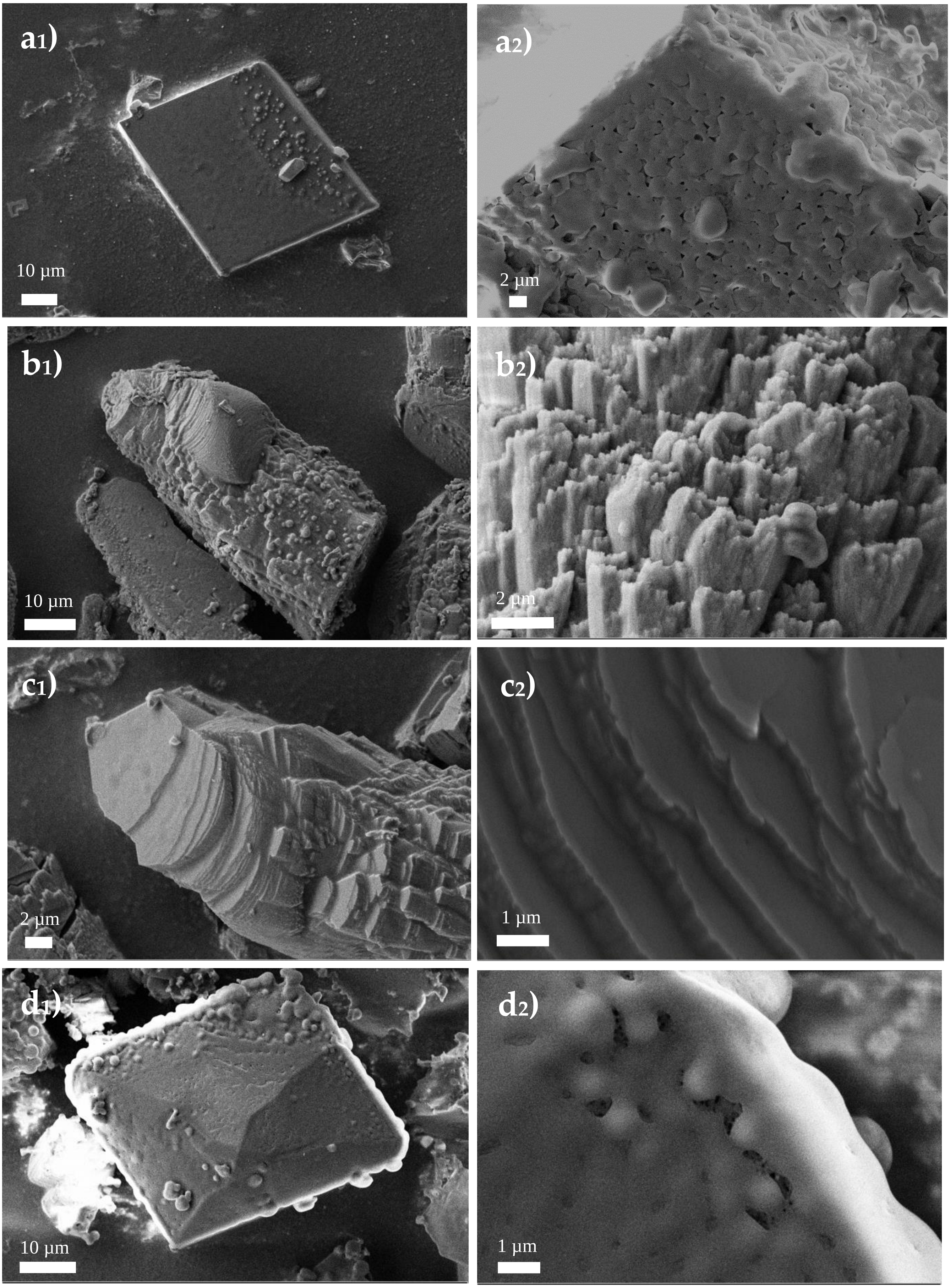

Figure S8. SEM images of $CaCO_3$ crystals grown in presence of MWCNT non-sonicated using macro-bridges: a_{1-2}) pristine MWCNT, b_{1-2}) MWCNT-Ox, c_{1-2}) MWCNT-IA and d_{1-2}) MWCNT-MMI.

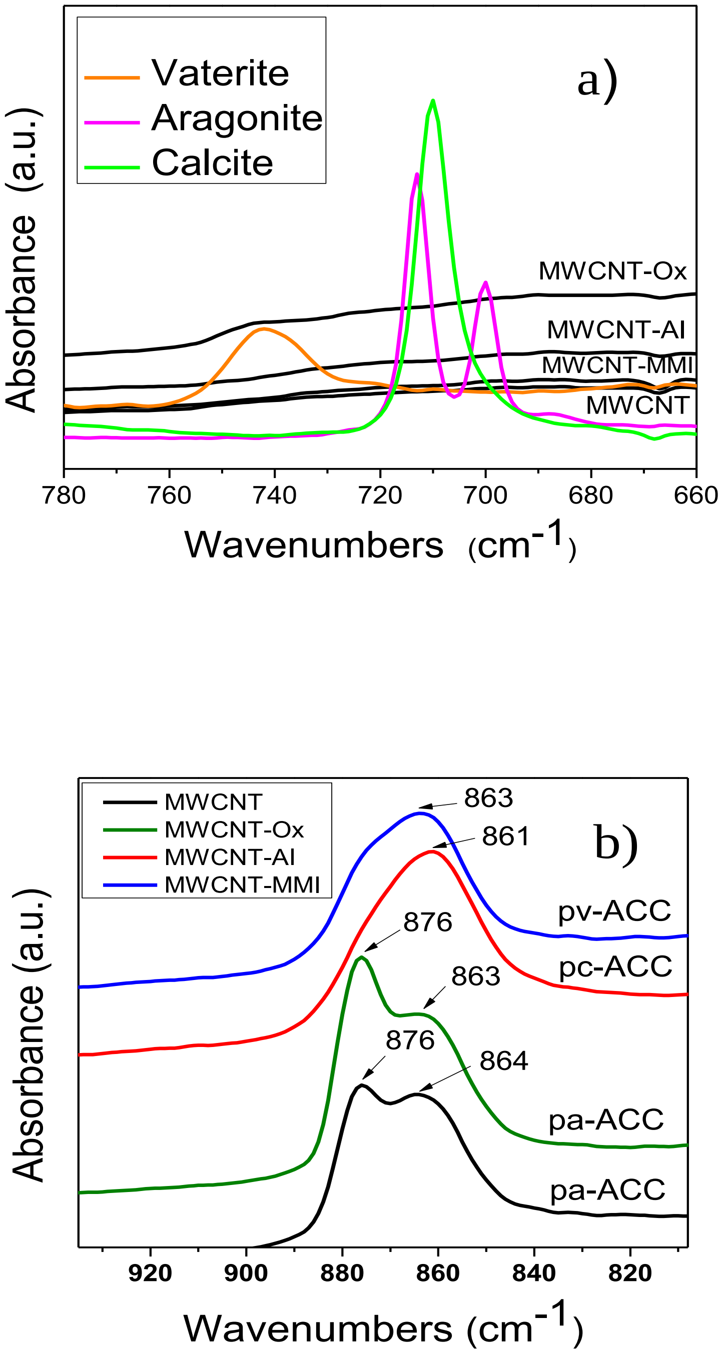

Figure S9. FTIR of ACC using MWCNTs additives. a) Crystalline spectral regions showing all spectral bands with all MWCNT additives and calcite, aragonite and vaterite polymorphism and b) Amorphous spectral region showing all ACC proto-structure stabilization.

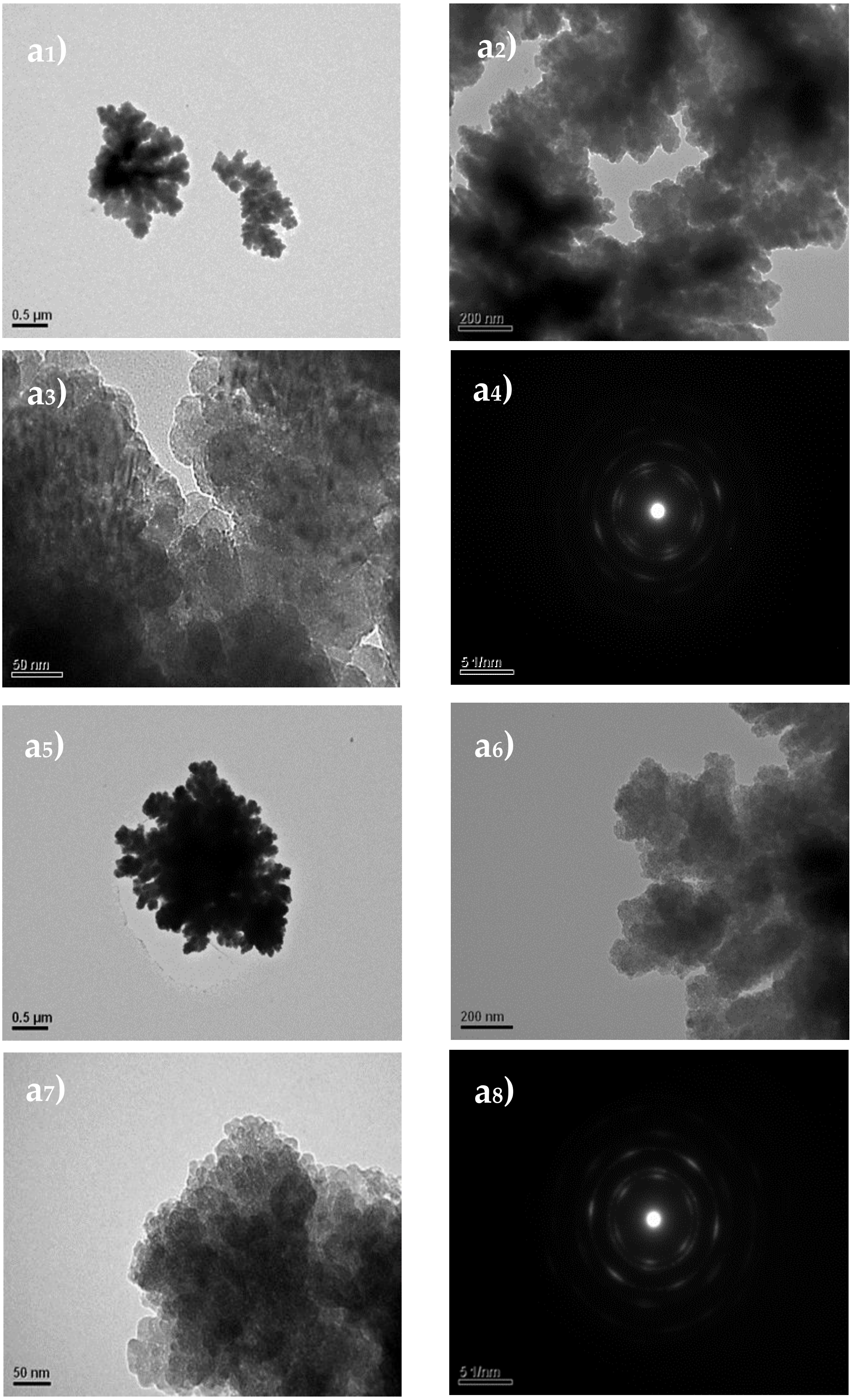
a1)
0.5 µm
a2)
200 nm
a3)
50 nm
a4)
5 1/nm
a5)
0.5 µm
a6)
200 nm
a7)
50 nm
a8)
5 1/nm

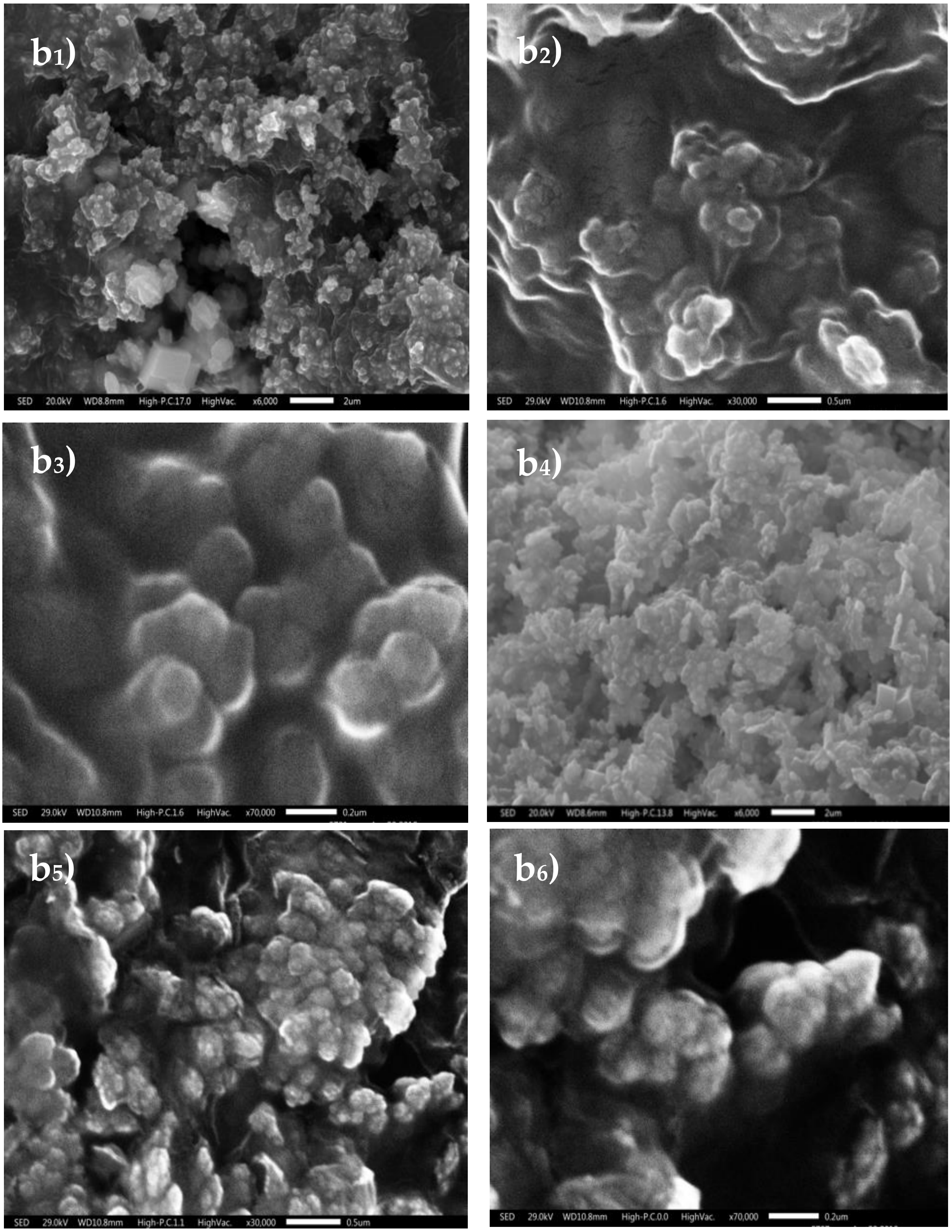

Figure S10. TEM images and electron diffraction (ED) patterns of ACC-proto structures and SEM images of $CaCO_3$ crystals grown in the presence of MWCNT and MWCNT-Ox before and post-nucleation points performed via PNC essays at pH 9.0, respectively. TEM and ED patterns using before nucleation point using a_{1-4}) pristine MWCNTs and a_{5-8}) MWCNT-Ox and SEM of post nucleation using b_{1-3}) MWCNTs and b_{4-6}) MWCNT-Ox.

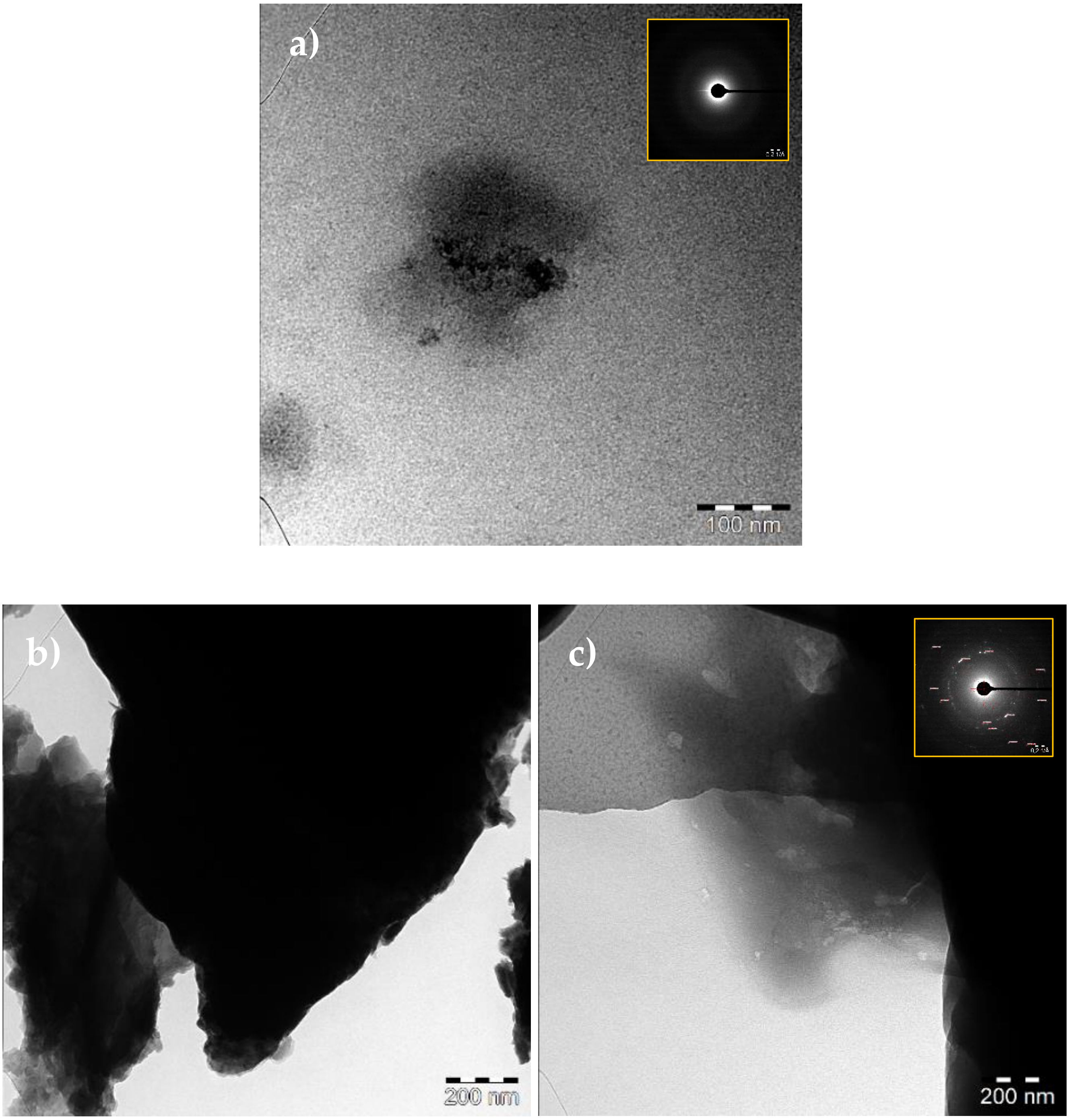

Figure S11. TEM images and electron diffraction (ED) patterns of ACC and $CaCO_3$ particles in the presence of pristine MWCNT. a) ACC particles and amorphous pattern (inset in Figure S11a), b) $CaCO_3$ particles and c) $CaCO_3$ particles after *in situ* burning showing less MWCNTs content and crystalline pattern (inset in Figure S11c).

Table S1. Zeta potential and half-width measurements of oxidized and functionalized MWCNTs obtained after 10 runs and sampling time of 128 μs without sonication.

Samples	Zeta potential (mV)	Half width (mV)
MWCNT	-1.62	5.19
MWCNT-Ox	-6.20	5.02
MWCNT-IA	-2.71	5.36
MWCNT-MMI	-1.89	3.87

Table S2. Effective diameter, half-width and polydispersity of oxidized and functionalized MWCNT obtained after 10 runs at elapsed time of 30 min after the third ultrasonication cycle in aqueous solution at pH 9.0.

Samples	Effective diameter (nm)	Half width (nm)	Polydispersity	Baseline index
MWCNT	5106.0	3871.9	0.575	0.0/94.12 %
MWCNT-Ox	1985.1	1172.1	0.349	0.0/92.53%
MWCNT-IA	1376.3	787.3	0.327	0.0/95.25%
MWCNT-MMI	1644.3	1036.3	0.397	0.0/92.25%

Table S3. Concentration of carboxylic acid groups of all MWCNTs determined by using acid-base titration.

Units of concentration	MWCNT	MWCNT-Ox	MWCNT-IA	MWCNT-MMI
mmol/g	1.8	3.7	15.0	12.0
